# Optical Fiber Sensors for High-Temperature Monitoring: A Review

**DOI:** 10.3390/s22155722

**Published:** 2022-07-30

**Authors:** Shaonian Ma, Yanping Xu, Yuxi Pang, Xian Zhao, Yongfu Li, Zengguang Qin, Zhaojun Liu, Ping Lu, Xiaoyi Bao

**Affiliations:** 1 Center for Optics Research and Engineering, Shandong University, Qingdao 266237, China; shaonianma@mail.sdu.edu.cn (S.M.); yuxi_pang@mail.sdu.edu.cn (Y.P.); zhaoxian@sdu.edu.cn (X.Z.); yfli@sdu.edu.cn (Y.L.); 2 Key Laboratory of Laser and Infrared System of Ministry of Education, Shandong University, Qingdao 266237, China; qinzengguang@sdu.edu.cn (Z.Q.); zhaojunliu@sdu.edu.cn (Z.L.); 3 School of Information Science and Engineering, Shandong University, Qingdao 266237, China; 4 National Research Council Canada, 100 Sussex Drive, Ottawa, ON K1A 0R6, Canada; ping.lu@nrc-cnrc.gc.ca; 5 Physics Department, University of Ottawa, 25 Templeton Street, Ottawa, ON K1N 6N5, Canada; xiaoyi.bao@uottawa.ca

**Keywords:** high-temperature measurement, fiber-optic sensors, blackbody radiation, fiber Bragg gratings (FBGs), crystal fibers

## Abstract

High-temperature measurements above 1000 °C are critical in harsh environments such as aerospace, metallurgy, fossil fuel, and power production. Fiber-optic high-temperature sensors are gradually replacing traditional electronic sensors due to their small size, resistance to electromagnetic interference, remote detection, multiplexing, and distributed measurement advantages. This paper reviews the sensing principle, structural design, and temperature measurement performance of fiber-optic high-temperature sensors, as well as recent significant progress in the transition of sensing solutions from glass to crystal fiber. Finally, future prospects and challenges in developing fiber-optic high-temperature sensors are also discussed.

## 1. Introduction

The reliable measurement of high temperatures plays a significant role in the aerospace field, metallurgical industry, and nuclear energy production [1,2,3,4,5]. For example, the application of long-range distributed high-temperature sensors guarantees the long-term safe operation of deep underground wells [6,7]. In the metallurgical industry, real-time measurement of the internal temperature of high-temperature boilers is key to monitoring combustion efficiency and safety prevention [1,8,9]. Temperature monitoring inside the combustion chambers and turbines of an aircraft or aero-engine can help extend its service life [2,10,11,12]. Extremely harsh environments with high temperatures, high pressures, and strong electromagnetic radiation present a challenge to traditional temperature sensors.

According to the installation and detection methods, high-temperature measurement technology can be mainly divided into contact measurement and non-contact measurement [3,13]. Thermocouple sensors made of precious metals are commonly used for contact temperature measurements thanks to their mature preparation process, ease of operation, wide temperature measurement range, and the capability for absolute measurements [14,15,16]. However, the thermocouple sensors have disadvantages of poor corrosion resistance, short service life, low measurement accuracy, and susceptibility to electromagnetic interference. Precious metal materials or alloys used to form thermocouple sensors are easily damaged at high temperatures, strong oxidation, or strong corrosion environments, affecting the service life and temperature measurement accuracy of thermocouples [17,18]. Infrared thermography (IRT) is representative of non-contact temperature measurement technology, which can avoid direct contact between temperature measurement equipment and high-temperature areas to achieve non-destructive testing [19,20,21]. Unfortunately, radiation temperature measurement technology is only suitable for surface measurements, such as explosion flame, and cannot detect the temperature of the internal structure of the closure device. Moreover, complex background noise is also a limiting factor in accurate temperature measurement [22].

**Figure 1:**
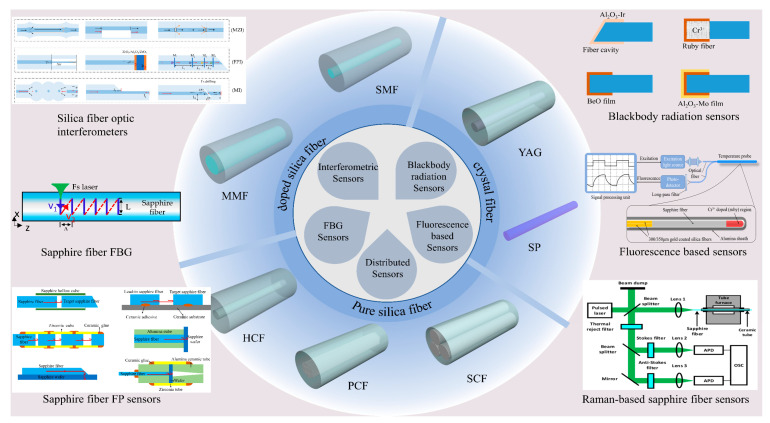
The temperature sensor classification, fiber type, and sensor mechanism from the inside to the outside. SP: sapphire, YAG: Yttrium aluminum garnet, SMF: single-mode fiber, MMF: multi-mode fiber, HCF: hollow-core fiber, PCF: photonic crystal fiber, SSCF: suspended-core fiber.

Compared to traditional electronic sensors [2], fiber-optic sensors have attracted intensive attention during the past decades due to their inherent advantages such as compact size, flexible structure, high sensitivity, high resolution, ability to multiplex, and immunity to electromagnetic interference [23,24,25,26]. In fiber-optic high-temperature sensing systems, various optical fibers are used as the sensor transducer, as the medium for data transmission, or both [27,28]. According to the temperature measurement principle, fiber-optic sensors can be divided into blackbody radiation sensors, fluorescence-based sensors, interferometric sensors, fiber Bragg grating (FBG) sensors, and distributed temperature sensors (DTS). The commonly employed high-temperature sensing fibers mainly include silica fibers and crystal fibers. Theoretically, the maximum temperature that a temperature sensor can withstand depends primarily on the fiber material rather than the sensing mechanism. Generally, silica-fiber-based temperature sensors are limited to operating within 1000 °C due to the diffusion of germanium dopant. In addition, temperature sensors based on pure silicon fibers (e.g., photonic crystal fibers, hollow-core fibers, suspended-core fibers) can operate at 1300 °C (near the melting point of silicon), and temperature sensors based on single-crystal fibers can operate stably below 1900 °C. Figure 1 shows the temperature sensor classification, fiber type, and sensor mechanism from the inside to the outside.

This paper will review the development of fiber-optic high-temperature sensors over the last 30 years, presenting their design and fabrication methods according to sensing type and typical temperature measurement performance. The full paper consists of eight sections. Section 1 is an introduction; Section 2 describes the development of the blackbody radiation sensors; Section 3 summarizes the fluorescent sensors based on various rare earth materials; Section 4 describes the interferometric sensor structures in silica and crystal fibers; Section 5 describes the fabrication of FBGs in silicon-based and crystal fibers; Section 6 describes the distributed sensing system based on Raman and Rayleigh scattering; Section 7 and Section 8 are the outlook and conclusion of this paper, respectively.

## 2. Blackbody Radiation Sensors

### 2.1. Sensing Principle of Blackbody Radiation

Blackbody optical fiber thermometers (OFT), created by sputtering a thin metallic or opaque oxide coating on the surface of the fiber to form a blackbody cavity, are mainly based on Planck’s law. When heated, the blackbody cavity will produce thermal radiation in an amount dependent on ambient temperature, the emissivity of the material, and the spectral range of detection. Temperature information can be easily obtained by measuring the intensity distribution or the spectral intensity.

According to Planck’s law, the radiation energy emitted by a blackbody per unit area and per unit wavelength is expressed as:

(1) $$E\left(\lambda ,T\right)=\frac{C_{1}}{\lambda^{5}\exp\left(\frac{C_{2}}{\lambda T}\right)-1}$$

where *λ* is the wavelength, *T* is the absolute temperature in Kelvin *K*. *C*_1_ *=* 2*hc*^2^ and *C*_2_ *= hc/k* (*c* velocity of light, *h* Planck’s constant, *k* Boltzmann constant) represent the first and second radiation constants, respectively. *C*_1_ *=* 3.7418 × 10^−16^ W·m^2^, *C*_2_ *=* 1.4388 × 10^−2^ m·K.

**Figure 2:**
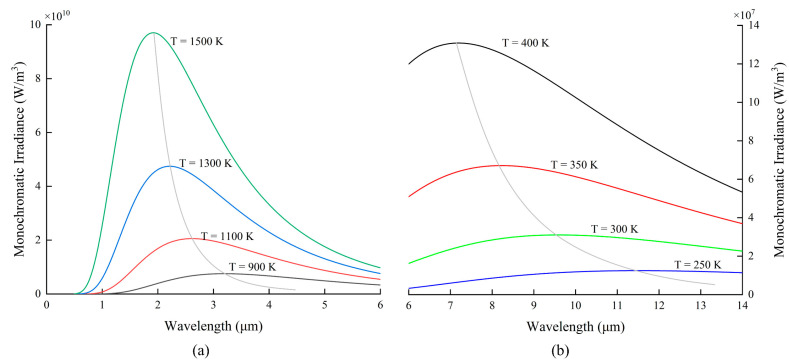
Planck’s law: electromagnetic radiation emitted by a blackbody in thermal equilibrium at a definite temperature. (**a**) High-temperature objects emit most of their radiation in the mid-wave infrared; (**b**) Low-temperature objects emit most of their radiation in the long-wave infrared.

According to Equation (1), the radiation spectrum distribution of an ideal blackbody at different temperatures and wavelengths is shown in Figure 2. It can be found that for the high-temperature range above 700 K, the radiation wavelengths are usually between 1 μm and 6 μm, as shown in Figure 2a. Optical fibers, with their transmission wavelength range (e.g., 0.35–2.4 μm for silica and 0.5–3 μm for sapphire fibers) falling exactly in the above band, are ideal sensing waveguides that are often used to make high-temperature sensors based on blackbody radiation.

When thermal equilibrium is reached, the optical radiation of the blackbody OFT is an explicit function of the cavity temperature. Integration of the light intensity detected by the photodetector gives the total radiated power in the detection wavelength range *λ*_1_–*λ*_2_.

(2) $$I\left(T\right)=\delta \cdot \sigma \left(\lambda \right)\cdot \int_{\lambda_{1}}^{\lambda_{2}}\varepsilon \left(\lambda ,T\right)\frac{C_{1}}{\lambda^{5}}{\left[\exp\left(\frac{C_{2}}{\lambda T}\right)-1\right]}^{-1}d\lambda$$

where *ε*(*λ*,*T*) represents the effective emissivity of the blackbody cavity, depending on the emissivity *ε* of the coating material and the ratio *g* = *D*/*L* of the cavity length *L* to the diameter *D*; *σ*(*λ*) represents the responsiveness of the photodetector; *δ* represents the transmission efficiency of the overall optical signal transmission system. Once the emissivity of the blackbody radiation sensor has been calibrated using a standard thermocouple sensor, it can be used for high-temperature measurements.

In practical applications, interference effects such as bending of the optical path, atmospheric effects, and low radiation intensities at lower temperatures can affect the effectiveness of the intensity measurement method. A two-color ratio measurement [29] based on Wien’s formula or a peak tracking method based on Wayne’s displacement law can effectively overcome these interferences.

Wien’s formula is a simplification of Planck’s law, as expressed in Equation (3), which can be used for values *λT* ≤ 3125 μm·K with an error of less than 1% and for values *λT* ≤ 2000 μm·K with an error of less than 0.1%.

(3) $$E\left(\lambda ,T\right)=\frac{C_{1}}{\lambda^{5}\exp\left(\frac{C_{2}}{\lambda T}\right)}$$

According to Equation (3), the measured signal *I_λ_*_,*n*_ of radiation intensities at *λ*_1_ and *λ*_2_ can be expressed as:

(4) $$I_{\lambda ,n}\left(T\right)=\frac{\delta \sigma \left(\lambda_{n}\right)\varepsilon_{n}}{\lambda_{n}^{5}\exp\left(\frac{C_{2}}{\lambda_{n}T}\right)}$$

where *ε_n_* is the monochromatic emissivity of the blackbody cavity, *n* = 1, 2.

By simplifying the ratio *R* = *I_λ_*_,1_/*I_λ_*_,2_ of the two signals measured, the ratio temperature *T_R_* of the two-color thermometer is obtained, as expressed in Equation (5), and *T_R_* will be the true temperature *T* when *ε*_1_ = *ε*_2_. Equation (5) shows that the two-color ratio method has the advantage that it is not affected by intensity fluctuations and that the temperature measurement can be achieved without knowing the surface emissivity. An error is mainly caused by unequal surface emissivities at two operating wavelengths of the blackbody cavity.

(5) $$T_{R}={\left(\frac{1}{T}+\frac{\ln\left(\varepsilon_{1}/\varepsilon_{2}\right)}{C_{2}\left(\lambda_{2}^{-1}-\lambda_{1}^{-1}\right)}\right)}^{-1}$$

The peak tracking method is based on Wayne’s displacement law which is a simplification of Wien’s formula:

(6) $$\lambda_{max}=\frac{2898}{T}$$

According to Equation (6), when the temperature rises, the emission peak *λ_max_* with the highest radiation energy drifts in the direction of the short wave, which is well illustrated in Figure 2a. Determining *λ_max_* at different temperatures enables temperature measurements to be made. This method usually uses an optical spectrum analyzer (OSA) or a photodetector to demodulate the signal. The principle of the OSA demodulation method is simple but expensive, and the temperature test range of the photodetector demodulation method depends on the responding wavelength range of the detector.

In general, blackbody radiation is more suitable for high-temperature measurements (*T* > 600 °C), while for lower temperatures (*T* < 600 °C), it becomes less accurate and precise.

### 2.2. Development of Blackbody OFT

Blackbody radiation fiber-optic temperature sensors are particularly attractive for applications where transient surface high-temperature measurements are required, such as explosive flames, rocket engine plume flames, gas turbine combustors, or high-temperature reactors [9]. The structure of the sensor, the selection of the blackbody cavity coating material, and the uncertainty analysis are the keys to the development of blackbody radiation high-temperature sensing technology.

**Figure 3:**
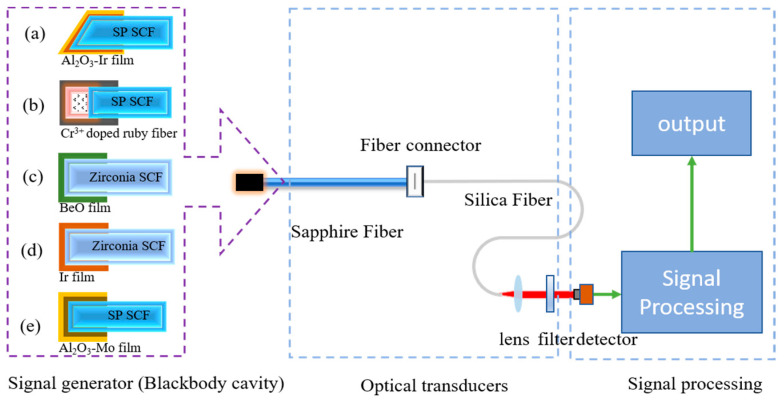
A schematic diagram for high-temperature blackbody optical fiber sensor system and various blackbody cavities made of (**a**) SP SCF + Al_2_O_3_-Ir; (**b**) SP SCF + Cr^3+^doped ruby fiber [30]; (**c**) Zirconia SCF + BeO; (**d**) Zirconia SCF + Ir; (**e**) SP SCF Al_2_O_3_-MO. SCF: single-crystal fiber.

A blackbody OFT does not require an additional light source and usually consists of three components: an optical signal generation system, an optical signal transmission system consisting of optical fibers, and an optoelectronic signal processing system, as shown in Figure 3.

For an optical signal generator as a radiation temperature conversion device, its structure, high-temperature resistance, radiation conversion rate, spectral detection range, etc., all profoundly determine the response speed, sensing accuracy, and temperature measurement range of the sensor [31,32]. Hence, the materials selection and design of the optical signal generator is the key to blackbody radiometry technology. In 1981, temperature measurement from 135 °C to 600 °C was achieved using a commercial section of silica fiber (20 cm long) as a thermal probe [33]. This method considers that the spectral composition detected by the detector is essentially determined by the highest temperature point along the fiber and is largely independent of the length of the thermal region. However, each location in a long fiber generates thermal radiation, and the temperature can vary widely [34], which may limit the flexibility and accuracy of the sensor.

Subsequently, in 1983, Dils first demonstrated a high-temperature blackbody OFT made of sapphire fiber [31], whose blackbody cavity is shown in Figure 3a. In this sensor, a thin metal or oxide coating (10 μm thick) is sputtered on the tip of a sapphire fiber (0.25–1.25 mm diameter, 0.05–0.30 m length) to form a blackbody cavity. A high-temperature sapphire fiber and a low-temperature glass fiber transmit the signal to a narrowband filter and an optical detector. Once the blackbody cavity emissivity is calibrated using a standard thermocouple, temperature detection can be achieved using the dual-wavelength method [29]. The use of sapphire fiber and metal coating easily breaks the 1000 °C temperature measurement limit of silica fiber and enables the sensor to measure temperature in the range of 600–2000 °C.

In 1999, in order to increase the temperature range of the blackbody radiation thermometer, Shen combined the blackbody radiation detection and fluorescence lifetime detection schemes [35,36] into the same system [30], as shown in Figure 3b. The sapphire fiber tip is doped with Cr^3+^ ions to form a blackbody cavity, and the output is coupled to a Y-shaped fiber bundle for signal transmission. A sandwich-type dual-frequency p-i-n detector is utilized to respond to both radiation and fluorescence. The sensor has a temperature range of 20 °C to 1800 °C and a good temperature response rate.

In addition to silica and sapphire fibers, other high-temperature resistant fibers are used to make high-temperature sensors. In 1999, Tong et al. developed a zirconia single-crystal fiber-optic sensor to improve the upper limit of work temperature measurement of the fiber-optic sensor [37]. In this sensor, a zirconia single-crystal fiber (SCF) was fabricated using the Laser Heated Pedestal Growth (LHPG) method, and a thin layer of bismuth oxide was sintered on the tip of the alumina fiber using a carbon dioxide laser to create a blackbody cavity, as shown in Figure 3c. Zirconia fiber (60 mm long), sapphire fiber (520 mm long), and silicon dioxide fiber together form the optical waveguide structure. The effective temperature measurement range of the sensor can reach 1200–2300 °C. However, compared to sapphire sensors, optical waveguides made of multiple optical fibers reduce the mechanical strength of the temperature measurement system as well as the measurement accuracy (4%).

Platinum (melting point 1700 °C) and iridium (melting point 2400 °C) are commonly used metal coating materials, but in the long-term high-temperature environment, the metal film is easily oxidized, leading to blackbody cavity performance instability or even life-shortening of the cavity. In 2015, Wei et al. selected yttrium-stabilized zirconia, which can be used at temperatures exceeding 2300 °C, as a cavity ceramic material to improve the long-term stability of the cavity, as shown in Figure 3d [38]. In 2017, Guo et al. fabricated a blackbody cavity by magnetron sputtering metal Mo on the end face of sapphire and then used Al_2_O_3_ as a protective film to achieve accurate measurements from 900–1880 °C, as shown in Figure 3e [39]. The sensor achieves high sensitivity absolute temperature measurement by integrating the radiation intensity between 1.4 and 1.5 μm with a maximum temperature deviation of 5°C.

As the temperature measurement area is relatively large, part of the extended fiber in the blackbody cavity also generates self-emission due to exposure to high temperatures, which can cause measurement errors [40,41]. In 2002, David G. Barker reported two possible methods to eliminate the effects of fiber self-emissions [42]. The first method is dual fiber thermometry, where a fiber with a reflective coating without a blackbody cavity is placed parallel to the blackbody thermometer to eliminate errors. The second method is spectral remote sensing. The intensity of the fiber self-radiation is measured with visible and infrared spectra, and then the temperature profile along the fiber is reconstructed. Compared with the second method, the two-fiber OFT is simpler, more efficient, and easier to implement. In addition, in 2003, they proposed a method to infer the temperature of the fiber tip by the spectral intensity of the emission at multiple wavelengths, hoping to solve the problem of fiber self-radiation [34]. Experiments have shown that the tip temperature measured by this method is more accurate than the values obtained using the standard two-color method [29] and may even predict the temperature distribution along the fiber.

In addition, scholars have explored research into sourceless high-temperature sensors based on blackbody radiation. In 2016, Tian et al. fabricated a sourceless fiber-optic high-temperature sensor using thermal radiation from the measurement environment itself as the light source, combined with a sapphire Fabry–Perot interferometer [41]. Interference spectra are generated when a source of ambient thermal radiation is directed into the interferometric cavity, and temperature measurements are achieved by processing the interference fringes. The sensor is cost-effective, compact, and has a resolution of 1 °C at 1593 °C. However, the weak radiation intensity at low temperatures limits the measurement range, and the weak coupling efficiency from the blackbody cavity to the end face of the fiber reduces the signal-to-noise ratio of the system. In 2019, Lei carried out theoretical and experimental research to improve coupling efficiency [43], building on Tian’s work [41]. It is proposed that the coupling efficiency can be effectively improved by using tapered fibers with a larger receiver aperture instead of end-flat fibers. Simulations and experiments have demonstrated that the coupling efficiency can be improved by approximately 38% using a 27.4 ° tapered fiber. In 2021, Chen et al. investigated the effects of low signal-to-noise ratio and blackbody radiation spectrum on interferometer sensing signals and suggested that the demodulation accuracy of the signal could be improved by increasing the temperature and removing the blackbody radiation envelope [44].

The sensing characteristics of the above-mentioned sensors are summarized in Table 1. The inability to perform lower temperature measurements is an important limitation of blackbody radiation high-temperature sensors. Although Cr^3+^-doped sapphire fibers have been proposed to improve sensing response in low-temperature environments, most blackbody cavity-based sensors still have difficulty detecting temperatures below 500 °C. Moreover, overcoming the high-temperature oxidation of metal films and improving cavity life remain important research objectives for their future commercialization.

## 3. Fluorescence-Based Sensors

**Figure 4:**
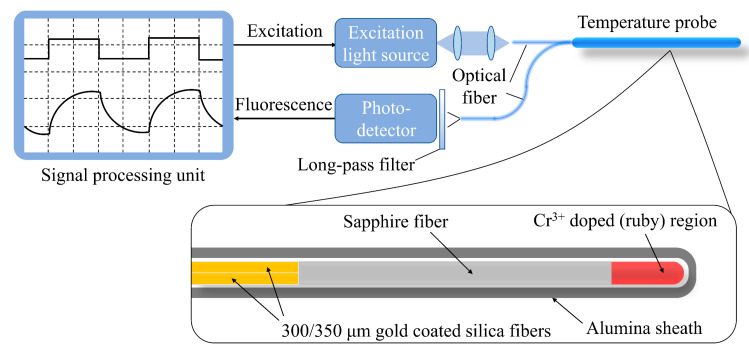
Structure of the fluorescence lifetime sensor with Cr^3+^-doped tip (ruby). (Adapted with permission from Ref. [45]).

Fluorescence-based high-temperature sensors are generally realized by attaching various photoluminescent materials to a silica fiber or a single-crystal fiber tip such as yttrium aluminum garnet (YAG), sapphire, and MgAl_2_O_4_ by the LHPG method or co-precipitation method. Figure 4 shows the schematic of the Cr^3+^-doped fluorescent sensor [45]. When a strong laser pulse hits the fluorescent material at the probe, it generates fluorescence, which gradually decays over time (also named fluorescence lifetime). The fluorescence lifetime and fluorescence intensity of the material depends on the external temperature and generally have a linear relationship [46]. At present, two commonly used fluorescence high-temperature sensing schemes are available: one is the fluorescence lifetime (FL) method [46,47,48,49,50,51,52,53,54] and the other is the fluorescence intensity ratio (FIR) method [55,56,57,58].

The FIR method achieves temperature testing by detecting the fluorescence intensity ratio of two wavelengths at different excited states. Compared to single wavelength fluorescence intensity testing, the FIR method avoids the effects of light source intensity fluctuations. However, it suffers from some limitations in terms of performance, such as poor linearity between temperature and intensity [49]. On the other hand, the FL method does not require precise measurement of output light intensity and is not affected by fluctuations in light source intensity or external background noise, so it is widely used in commercial sensors.

Fluorescence lifetime methods have been used for sensing measurements since the 1980s, but not in fiber form [59]. With the development of optical fiber technology, optical-fiber-based fluorescent temperature sensors have been widely studied. When silicon is used as the sensor waveguide, the sensor usually operates below 400 °C. The sensor can reach a measurement value of approximately 600 °C by using a metal-coated optical fiber [60]. Later, single-crystal optical fibers were widely used in fluorescent sensors due to their higher melting point (>2000 °C), excellent mechanical properties, and extreme resistance to oxidation. In addition to the waveguide, the temperature measurement range of the sensor is also affected by the fluorescent material. Table 2 summarizes the temperature sensing performance of fluorescence-based sensors with different doping materials in recent years. It can be found that the fluorescent material not only determines the upper limit of the measurable temperature of the sensor but also determines the sensitivity, stability, and strength of the signal under detection. For example, the burst effect of Er^3+^-doped YAG above 600 °C causes rapid decay of fluorescence intensity and fluorescence lifetime, resulting in different sensitivity of the sensor in different temperature intervals [61]. Tm-doped Y_2_O_3_ has a weak fluorescence intensity, so the signal-to-noise ratio of the detected signal is poor and not suitable for high-precision measurements [62]. Therefore, the development of fluorescent sensors with high luminescence intensity and good linearity is a challenge to be faced in the future.

## 4. Interferometric Sensors

Fiber-optic interference sensors are widely used for measuring temperature, strain, pressure, refractive index, and other parameters because of their simple principle, high accuracy, linear response, and easy manufacturing [63]. When wide-spectrum light hits a typical optical interferometer, the presence of the optical path difference (OPD) causes multi-beam interference to form a periodic pattern, known as an interference pattern. The output intensity (*I*) of the two-beam (*I*_1_, *I*_2_) interference of the fiber interferometer is expressed as:

(7) $$I=I_{1}+I_{2}+2\sqrt{I_{1}I_{2}}\cos\left(\frac{2\pi }{\lambda }\cdot \mathrm{OPD}+\varphi_{0}\right)$$

where *λ* is the wavelength of the light beams; *φ*_0_ is the initial phase of the interferometer (normally equals to zero).

The OPD varies with the external temperature, so temperature measurement can be achieved by calculating the value of the OPD from the interference spectrum [64,65]. When the temperature is below 1000 °C, Mach–Zender interferometer (MZI), Fabry–Perot interferometer (FPI), and Michelson interferometer (MI) based on silica fiber are widely used for high-temperature sensing. Various silica fibers have been used to make high-temperature interference sensors, such as standard single-mode fiber (SMF), multimode fiber (MMF), thin-core fiber (TCF), dispersion compensation fiber (DCF), holey fiber (HF), hollow-core fiber (HCF), multicore fiber (MCF), microfiber (MF), and photonic crystal fiber (PCF). Due to the reduced mechanical strength and thermal diffusion of the germanium dopant, silica fiber-based temperature sensors typically cannot withstand temperatures exceeding 1000 °C [66,67]. In addition, FPI and MI fabricated on sapphire fiber enable temperature measurements above 1000 °C. In this section, the current status of research into silicon-based and SCF-based interferometric sensors will be presented, respectively.

### 4.1. Silica Fiber-Optic Interferometers for High-Temperature Sensing up to 1000 °C

With the advancement of microfabrication technologies such as carbon dioxide lasers and femtosecond (fs) lasers, various micro-structured fiber-optic interference sensors have been fabricated, and their more compact structures offer the possibility of sensor packaging and practical applications [26]. These interferometric sensors are divided into transmissive types, such as MZI, and reflective types, such as FPI and MI. Alternatively, these interferometric sensors can also be categorized into two main groups based on the transmissive mode type involved in the light interfering process. One is core-mode coupling interferometers like FPIs, and the other is core-cladding-mode interferometers (CCIs), including both Michelson and Mach–Zender configurations [68].

**Figure 5:**
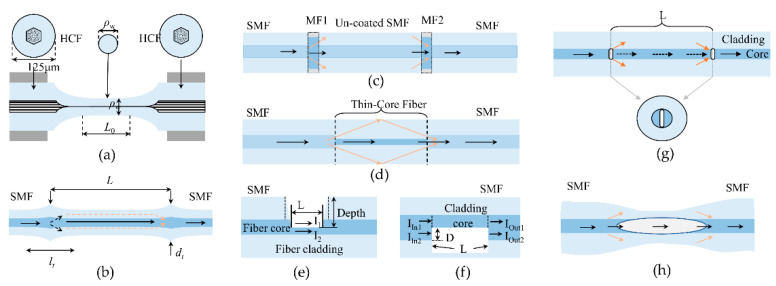
Typical types of MZI sensors for high-temperature measurements realized with optical fiber tapers (**a**,**b**), core diameter mismatch (**c**,**d**), trench structure (**e**,**f**), laser micromachining (**g**,**h**): (**a**) MZI consists of a holey fiber taper with collapsed air holes in the waist [69]; (**b**) MZI consists of two waist-enlarged fiber bitapers in a circular step-index SMF; (**c**) MZI by splicing MMF-SMF-MMF; (**d**) MZI by splicing SMF-TCF-SMF; (**e**) MZI consists of a side-ablated structure on the SMF; (**f**) MZI by removing part of the fiber core and cladding by fs laser micromachining; (**g**) MZI by concatenating two microcavities separated by a middle section; (**h**) MZI by drawing SMF with hollow sphere into microfiber by use of flame brushing technique.

MZI typically uses a variety of microstructures as beam splitters and beam combiners to separate and combine transmitted light to achieve double-beam interference. As shown in Figure 5, according to the production method, MZIs can be divided into tapered-fiber-based MZI [69,70], core diameter mismatch-based MZI [71,72], trench structure-based MZI [73,74], laser micromachined-based MZI [75,76], etc. Although they have good sensing performance, MZI-based sensors are less suitable for measurements in narrow and enclosed spaces than reflective sensors.

**Figure 6:**
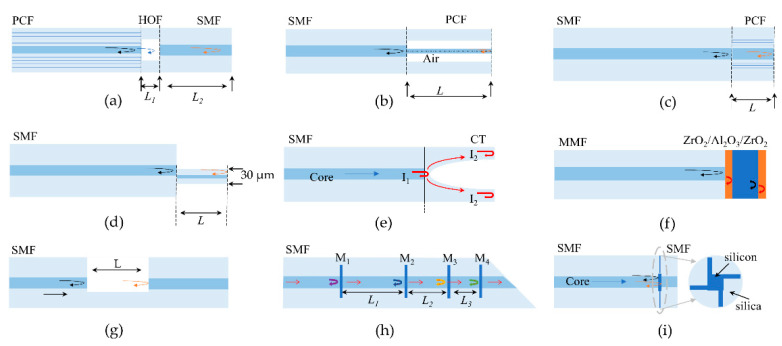
Typical types of FPI sensors for high-temperature measurements realized with splicing technology (**a**–**e**), coating dielectric film (**f**), laser micromachining (**g**,**h**), silicon layer integration (**i**): (**a**) FPI by fusion splicing a 70 μm HOF and a 510 μm SMF at the end of a PCF [77]; (**b**) FPI by splicing a special PCF (MM-HNA-5) to an SMF; (**c**) FPI by splicing an SMF to a short PCF; (**d**) FPI by splicing an SMF to a short MF; (**e**) FPI by splicing a concave-shaped HCF to an SMF; (**f**) FPI by depositing three layers of ZrO_2_/Al_2_O_3_/ZrO_2_ dielectric materials on the tip of an MMF; (**g**) FPI by micromachining a micro notch in an SMF by fs laser; (**h**) FPI based on four in-fiber mirrors fabricated by fs laser; (**i**) FPI by completely embedding a thin (<1 μm) a-Si:H layer within an SMF.

FPI is another popular high-temperature sensor, usually achieved by creating two reflective mirrors along the fiber. As shown in Figure 6, high-temperature FPI sensors based on silica fiber are usually achieved by splicing two different fibers [77,78,79,80,81], depositing dielectric films on fiber tip [82], fs laser micromachining [83,84,85], or silicon layer integration [86]. For high-temperature FPI sensors with optical fiber splicing, the fiber end face can easily be contaminated or even damaged in harsh environments [87]. Therefore, good encapsulation protection is required for high-temperature measurements. The fabrication of in-fiber mirrors with fs lasers overcomes these problems, but at the same time, increases the production costs.

**Figure 7:**
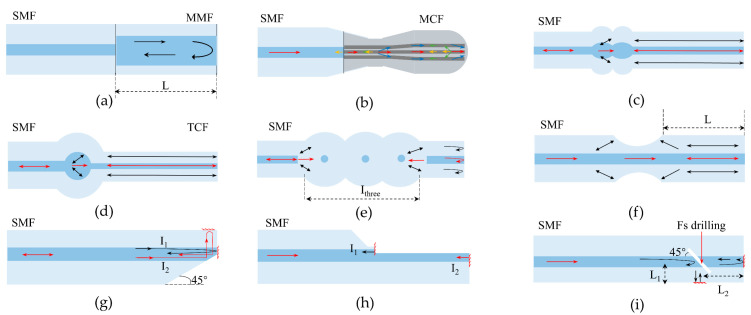
Typical types of MI sensors for high-temperature measurements realized with core diameter mismatch (**a**,**b**), silicon-microsphere (**c**–**f**), polishing technique (**g**), laser micromachining (**h**,**i**): (**a**) MI by fusion splicing an MMF to an SMF [88]; (**b**) MI by splicing an SMF to a segment of MCF with a taper region; (**c**) MI based on a peanut-shape fiber tapers made by fusion splicer; (**d**) MI by fusion splicing a bi-taper in TCF; (**e**) MI by fusion splicing a three-microsphere-structure (TMS) in SMF; (**f**) MI by form a taper in SMF; (**g**) MI by fabricating a 45°angle at the end face of SMF by polishing technique; (**h**) MI by cutting a step structure into the fiber at its tip by fs laser; (**i**) MI based on an inclined narrow slit inside SMF.

Compared to FPIs or MZIs, MIs have higher temperature sensitivities due to their long interferometer length. High-temperature fiber-optic sensors based on MI are usually fabricated in two ways, as shown in Figure 7. One is to couple part of the energy of the core mode into the cladding by fusing two fibers with mismatched core diameters [88,89,90,91] or silicon-microspheres [92,93,94,95,96,97,98,99]. The two optical paths transmitted along the core and the cladding of fiber are reflected by the mirror and recombined to achieve double-beam interference. The other is to create a smooth reflective surface directly on the single-mode fiber by polishing techniques [100] or femtosecond lasers [101,102] to separate and combine the input optical signal. Similarly, the protection of the MI sensor’s packaging and the difficulty of mass production are issues that need to be overcome for its commercial application.

Table 3 summarizes the silicon-based fiber-optic interferometric high-temperature sensors. In general, silica fiber-optic interference sensors have good temperature measurement accuracy. However, the melting point of silica material limits its upper-temperature measurement limit. Especially for long-time high-temperature measurements, the maximum temperature measurement capability of these systems is limited to less than 1200 °C. Therefore, the use of other high-temperature resistant optical fibers to make fiber-optic interference sensors is an important research direction for the future.

### 4.2. Sapphire FPI Sensors for High-Temperature Sensing

The large diameter and highly multimode nature of sapphire inhibit the migration from silica fibers to sapphire fibers in conventional interferometry [103]. Over the years, researchers have initially realized the fabrication of sapphire FPI using different fiber fusion [104,105,106,107,108], sapphire wafer fusion [109,110,111,112], and metal coating [113,114,115,116]. Fabry–Perot interferometric sensors can be divided into two main groups: intrinsic FPI (IFPI)and extrinsic FPI (EFPI) [63]. Based on the thermo-optical and thermal expansion effects, the refractive index and thickness of the Fabry–Perot (FP) cavity change with temperature, respectively, thus affecting the OPD of the FPI and the temperature measurement can be achieved by retrieving the values of OPD.

In 1992, Wang et al. first spliced sapphire fiber to silica fiber to produce intrinsic FPI [105]. Limited by the melting point of silica, the achieved temperature measurement range is only 310–976 °C with a resolution of 0.2 °C. Compared with silica fiber, sapphire fiber has a larger mode field diameter and the effect of phase dispersion, so the parallelism of the reflective surface has a greater impact on the visibility of interference fringes. Wang et al. subsequently improved the stripe visibility from 0.18 to 0.41 by optimizing the structure and fusion method [104]. They investigated the effect of lateral offset and longitudinal gap on the visibility of interference fringes at the splice of the two fibers, respectively. The results show that streak visibility can be improved to a maximum of 0.41 when lateral offset and longitudinal gap are controlled within the range of ~10 and ~25 μm, respectively. Although the method enables temperature measurement with an accuracy of 0.1 °C, the complex fabrication process limits the possibility of mass production.

**Figure 8:**
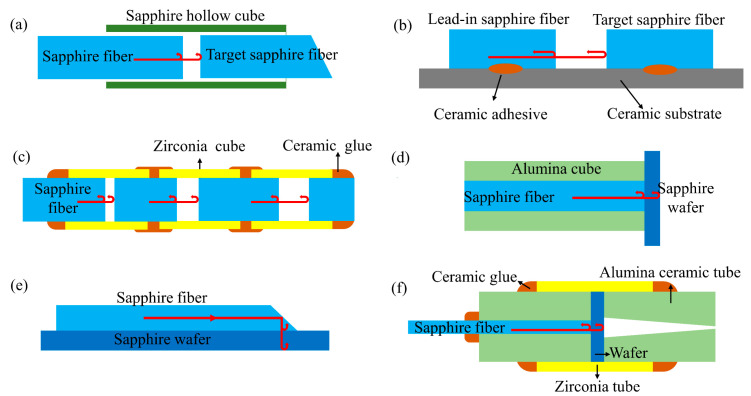
Configuration of various types of Sapphire FPIs. (**a**) Air cavity EFPI formed by two sections of sapphire fiber [106]; (**b**) EFPI formed by fixing two sapphire fibers on a ceramic base; (**c**) Multiplexed high-temperature sensor by concatenating multiple air-gap sapphire FPIs; (**d**) FPI formed by bonding a section of sapphire fiber with a sapphire wafer end-to-end; (**e**) Sapphire wafer is flush-mounted to sapphire fiber to form FPI; (**f**) EFPI with high-temperature resistant package.

EFPI sensors usually consist of multiple components to form an air cavity for high-temperature or strain measurement at high temperatures. In 1995, Wang et al. placed two sections of sapphire fiber end-to-end to form an air cavity (as shown in Figure 8a) and achieved a small-scale temperature-strain dual-parameter test [106]. The EFPI sensor has a 3.5% temperature resolution in the 150–650 °C range. Subsequently, in 2003, they made an EFPI by fixing two sapphire fibers on a ceramic base (as shown in Figure 8b) and achieved strain measurement at 1004 °C [107]. In 2010, Wang et al. fabricated a multiplexed high-temperature sensor by concatenating multiple air-gap sapphire FPIs [108]. Multiple segments of sapphire fibers were bonded to zirconia tubes and glued together with high-temperature ceramic adhesive. FPIs with different OPDs were fabricated by controlling the spacing between the sapphire fibers at both ends. The sensor structure is shown in Figure 8c. However, this structure still requires a high degree of parallelism between the end surfaces of two fiber sections, which makes the fabrication difficult.

In order to reduce the fabrication difficulty of EFPI, in 2005, Zhu et al. reported a fiber-optic high-temperature sensor with a sapphire fiber as the optical waveguide and a sapphire wafer as the sensing element [109]. The sensor achieves temperature measurement from 230 to 1600 °C with 0.2% accuracy by detecting the interference spectrum of the two reflective surfaces of the sapphire wafer, as shown in Figure 8d. In 2006, Zhu et al. proposed a new flat-paste method to improve the coupling efficiency between sapphire fibers and sapphire wafers [110]. As shown in Figure 8e, this method connects a 45° tangent sapphire fiber to the surface of a sapphire wafer to make a high-temperature fiber-optic sensor, which is more conducive to the transmission of light on the coupling surface. In addition, this method does not require an aluminum oxide tube as a support structure, so there is no measurement error due to different expansion coefficients of multiple components. The experimental results show that the sensor has a resolution of 0.4 °C at 1170 °C. Compared to sapphire fiber, sapphire wafers have good surface quality and thermal dependence and are easy to fabricate in bulk.

However, the sapphire wafer itself is susceptible to contamination, which reduces the stability of the interferometer. In 2020, Wang et al. encapsulated EFPI sensors in alumina ceramic tubes, as shown in Figure 8f, and the method successfully suppressed airborne dust contamination to improve the stability of the sensors [117]. The wavelength–temperature relationship was investigated in the temperature range of 25–1550 °C during the ramp-up and ramp-down process. In addition, the Fourier variation method was used to calculate the OPD to achieve temperature measurements and several repeatability experiments were carried out. The experimental results show that the method has good stability and accuracy, with a sensitivity of 32.5 pm/°C at 1550 °C. Therefore, it has some potential for practical application.

In recent years, air cavity EFPIs have been reported and successively applied for high-temperature sensing. The method is to etch the sapphire wafer [111,112] or sapphire fiber [118] first and then bond it directly with the sapphire wafer to form an air cavity. In 2017, Li et al. proposed a direct sapphire bonding method using plasma surface activation, hydrophilic prebonding, and high-temperature annealing [111]. In 2019, they fabricated another EPFI sensor for dual parameter measurements of temperature and pressure using directly bonded double-layer sapphire wafers [112]. The sensor is stable at 800 °C and 20 kPa, and the temperature coefficient of the cavity length changes and pressure-sensitivity changes are 1.25 nm/°C and 0.00025 nm/(kPa·°C), respectively. However, the use of high-temperature adhesives limits the temperature range of this sensor.

**Figure 9:**
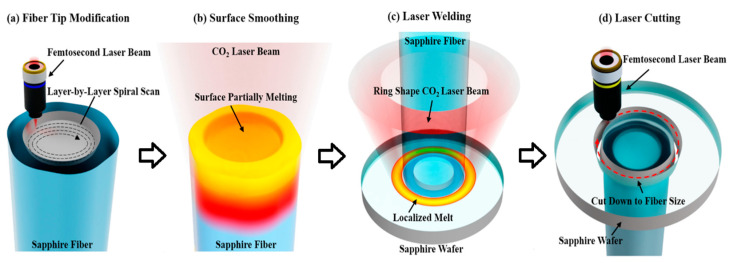
Schematic diagram of the all-sapphire FPI fabrication process. (Reprinted with permission from Ref. [118]). (**a**) Machining a 12 μm deep pit with 90 μm diameter on the end face of a sapphire fiber using fs laser; (**b**) Hydrofluoric acid immersion and CO_2_ laser heating to reduce the reflective surface roughness; (**c**) Laser welding technique to weld a sapphire wafer of 30 μm thickness and 350 μm diameter to the fiber; (**d**) Femtosecond laser excision of excess sapphire wafer.

To overcome the temperature limitations of high-temperature ceramic adhesives, an all-sapphire fiber-optic microsensor was successfully fabricated in 2020 [118]. In this sensor, interferometric cavity fabrication was successfully realized at the tip of 125 μm sapphire fiber by femtosecond laser micromachining and CO_2_ laser welding. The FP cavity fabrication process is shown in Figure 9. Experimental results show that the sensor has good temperature measurement performance at 1455 °C with a temperature response factor of 1.32 nm/°C and a resolution of 0.68 °C. The method is effective in avoiding crosstalk generated by strain due to thermal expansion mismatch of different materials and adhesive degradation. However, the use of various microfabrication techniques raised the production cost.

In addition to the sensors made of sapphire wafers mentioned above, the deposition of metal oxide films on the tips of polished sapphire fibers has also been widely studied. In 2003, Cheng et al. successfully fabricated FPI temperature sensors by using chemical vapor deposition (CVD) to grow 0.5–2.0 μm thick SiC films on sapphire fiber tips [119]. Although the temperature measurement range was only 22–540 °C, it nevertheless proved the feasibility of making thin-film FPI. Subsequently, Wang et al. successfully prepared metal oxide thin-film FPI sensors by depositing a layer of Ta_2_O_5_ and ZrO_2_ films, respectively, on the tip of a sapphire fiber using electron beam evaporation [113,114]. The inherent parallelism and smoothness of the Ta_2_O_5_ films are well suited for low coherence temperature measurements. Temperature measurements from 200–1000 °C with a temperature resolution of 1.4 °C were achieved by calculating the wavelength shift of the interference spectrum [113]. In addition, the multilayer coating method of the sapphire tip was also reported [115,116]. In 2015, Huang et al. demonstrated a low-cost temperature sensor fabricated from a ZrO_2_/Al_2_O_3_/ZrO_2_ (ZAZ) triple-layer film structure [115]. In this work, they prepared FPIs with thicknesses of 283 nm, 1396 nm, and 283 nm from ZrO_2_, Al_2_O_3,_ and ZrO_2_ (ZAZ), respectively, using physical vapor deposition (PVD) techniques. The refractive index difference between ZrO_2_ and Al_2_O_3_ facilitates the strong reflection of light at the coupling surface for interferometric spectrum measurements. The temperature measurement is achieved by detecting the wavelength shift around 480 nm using the peak tracking method. The sensor has a sensitivity of 1.8 × 10^−5^/°C between 100.9 °C and 1111.0 °C. In summary, metal thin film FPI sensors have the advantages of low cost, mass production, small size, and embedded measurement. However, when the temperature is higher than 1200 °C, the film is easily oxidized and broken, so it is not suitable for long-term high-temperature measurement.

**Figure 10:**
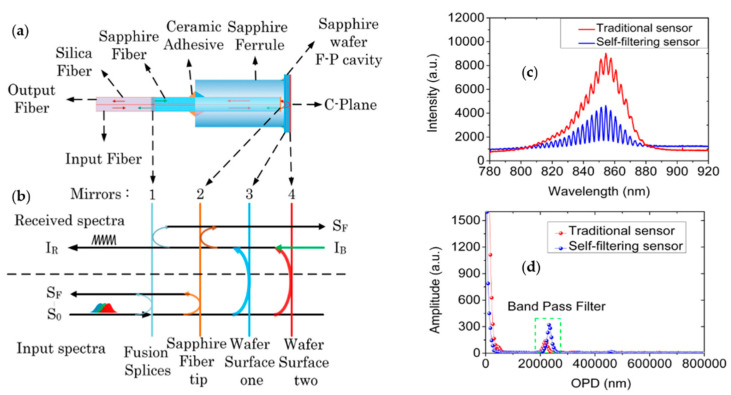
The schematic diagram and the test results of self-filtering sensor. (Reprinted with permission from Ref. [120]). (**a**) Schematic of self-filtering sensor; (**b**) Optical transmission path of self-filtering sensor; (**c**) Interference spectra of a conventional sensor and the self-filtering sensor at 1000 °C; (**d**) Frequency domain spectrum of the two sensors.

To improve stripe visibility and signal-to-noise ratio (SNR), in 2019, Yu et al. proposed a self-filtering high-resolution EFPI sapphire fiber sensor [120]. In the sensor, two well-polished sapphire fibers are arranged side by side to achieve shunt transmission between background light (S_F_) and interference signal (I_B_) according to the fiber diffusion coupling theory. The sensor structure is shown in Figure 10a, where two identical sapphire fibers are spliced with a section of multimode fiber and the other end is connected to a sapphire wafer through a sleeve. The optical transmission path is shown in Figure 10b. In the first sapphire fiber, the input light S_0_ passes through the fusion point and the sapphire fiber end face to reflect the background light S_F_. The signal light passes through the two reflective surfaces of the wafer to produce an interference signal (I_R_). According to the fiber diffusion coupling theory, the interference signal is coupled into a second sapphire fiber and received by the spectrometer. I_B_ consists mainly of blackbody radiation and stray light, representing the DC term of the interference spectrum. By transmitting the input and output light to different channels, most of the DC components of the received signal spectrum can be effectively filtered out. Figure 10c,d shows the comparison of the test results between the conventional sensor and the self-filtering sensor at 1000 °C. It can be found that the self-filtering sensor removes noise significantly and has higher visibility of interference fringes (over 43.96%) and signal-to-noise ratio (over 20.14 dB).

Table 4 summarizes the various types of sapphire high-temperature FPI sensors and their performance. As mentioned above, the performance of sapphire FPI is strictly dependent on the precise fabrication of FP cavities, and a very small angular deviation (10^−2^ deg) still significantly reduces the streak visibility [121]. Although researchers are trying to improve FPI stripe visibility, most methods are complex and costly and not suitable for mass production. In addition, overcoming the blackbody radiation noise generated by sapphire and removing the use of high-temperature ceramic binders are also priorities for future research.

### 4.3. Sapphire Michelson Interferometric Sensors

**Figure 11:**
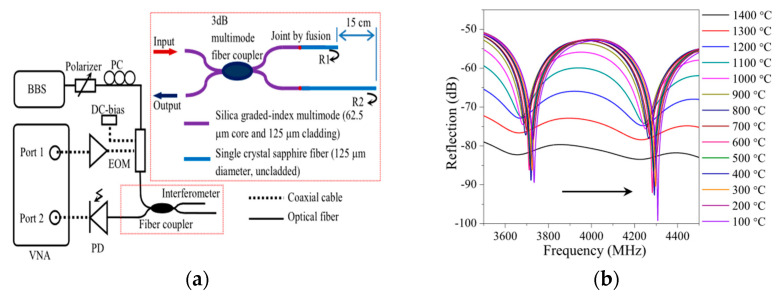
(**a**) Schematic of an OCMI interrogation system; (**b**) Interference fringes of the SP fiber-based OCMI at different temperatures ranging from 100 °C to 1400 °C. (Reprinted with permission from Ref. [122]).

Researchers have tried a variety of methods to reduce the impact of the multimode properties of sapphire fibers on interferometry. However, the use of various complex structures and micromachining devices limits the repeatability and reproducibility of the sensors. The dependence of the sensor temperature measurement performance on the parallelism of the F-P cavity remains, and the blackbody radiation generated by the sapphire is a source of noise that cannot be ignored. Therefore, in 2015, Huang et al. reported an optical carrier-based microwave interferometry (OCMI) method [122]. OCMI is a combined microwave and optical wave exploration, the essence of which is to read optical interferometer in the microwave field [123,124,125]. The schematic diagram of the OCMI system is shown in Figure 11, where a Michelson interferometer composed of sapphire fiber is added to a microwave network analyzer for the emission and detection of microwave signals. The OPD of the interferometer is smaller than the coherence length of the microwave source and larger than the coherence length of the light source. The optical carrier is established in a non-coherent manner, while the microwave signal (envelope) is established in a coherent manner, resulting in an interferogram in the microwave domain [125]. Therefore, the contribution of optical interference in the detection signal is almost zero, and temperature can be achieved using microwave interference. The results show that temperature measurements from 100–1400 °C can be effectively achieved for 4300 MHz microwave frequency monitoring. The sensor achieves an average temperature sensitivity of 64 kHz/°C with a standard deviation of 30 kHz. Experimentally, the OCMI system is expected to overcome the limitations of high precision fabrication requirements of sapphire fiber interferometer and background noise of blackbody radiation.

## 5. Fiber Bragg Grating Sensors

Fiber Bragg grating is an all-fiber passive device that reflects specific wavelengths, which is mainly used for sensing, communication, filtering, dispersion compensation, and making fiber lasers [126]. FBG is achieved by periodically modulating the refractive index along the fiber axis. When broadband-spectrum light hits the FBG, the light component that matches the Bragg wavelength *λ*_Bragg_ is reflected and other light components continue to transmit forward. The Bragg wavelength is mainly determined by the refractive index modulation period *ᴧ* and the effective refractive index of the fiber *n_eff_*, as expressed by the equation:

(8) $$\lambda_{\mathrm{Bragg}}=2n_{eff}ᴧ$$

When the temperature changes, a change in the refractive index of the fiber is induced, resulting in a linear drift of the Bragg wavelength. Temperature measurement can be easily achieved by demodulating the Bragg wavelength variation. Compared with other fiber-optic sensors, FBG-based sensors have the advantages of high stability, good linearity, multiplexing capability, mass production, and other characteristics, which have been widely used in industrial production. At present, there are two main methods for fiber grating fabrication, one of which is Ultraviolet (UV)-induced technology, and the other is femtosecond laser inscription technology.

The UV laser-induced technique is highly dependent on the optical fiber core photosensitivity and is suitable for fabricating FBGs in germanium-doped silica fibers. Typically, type I FBGs made by UV laser phase masks operate at temperatures below 450 °C, and erasure occurs at higher temperatures [24]. The regenerated grating formed after annealing can sustain high temperatures up to 1295 °C. However, the mechanical strength of the fiber is significantly reduced, which makes the sensor not suitable for practical application [127]. In addition, there are also some non-standard FBGs, such as surface relief fiber Bragg gratings (SR-FBGs) [128] or helical long-period fiber gratings (HLPFGs) [129], which change the physical feature of the fiber and are therefore not subject to refractive index high-temperature erasure and are suitable for high-temperature measurements at 1100 °C. Similarly, this approach reduces the mechanical strength of the sensor, so it is not suitable for extreme environmental applications.

In contrast, femtosecond laser inscription technology can fabricate FBGs in non-photosensitive fibers such as pure silicon fibers [130] or sapphire fibers [131] with the highest operating temperature at 1300 °C or 1900 °C, respectively. In this section, FBGs made by femtosecond laser on various fibers will be reviewed according to the FBG formation method.

### 5.1. Silica FBGs for High-Temperature Sensing up to 100 °C

Femtosecond lasers have extremely high peak power and ultra-short pulse widths, so they are suitable for refractive index modulation of fiber waveguides [24]. When the energy from the fs laser converges to the fiber, a large amount of electron-ion plasma is generated at the focus of the fiber according to the nonlinear absorption effect, resulting in material compaction or collapse and permanent modulation of the refractive index. Three different refractive index modulations are produced depending on the exposure intensity, namely isotropic refractive index increase [132,133], anisotropic refractive index change (birefringence) [134,135], and negative refractive index modulation (voids) [133]. When the exposure intensity is below the damage threshold of the medium, melting and subsequent rapid solidification will occur at the focal point, forming a type I FBG. At very high energies, permanently damaged cavities are created within the fiber, forming a more thermally stable type II FBG. They have a different annealing behavior and grating structure [24,136].

In 2003, Mihailov et al. first realized FBG fabrication using an fs laser phase mask technique [137]. Similar to the conventional UV laser phase mask technique, they successfully fabricated a high-reflectivity FBG using an 800 nm, 120 fs laser in a standard germanium-doped communication fiber (Corning SMF-28). Moreover, the FBG has good thermal stability and has not been erased at 300 °C for two weeks. Meanwhile, the FBG can be produced by multiple pulse exposures and the reflectivity increases linearly with the number of pulses. Subsequently, studies on the fabrication of FBGs by phase mask technique using UV- and IR-emitting fs laser sources were reported successively [138,139,140]. FBGs etched with the femtosecond laser phase mask technique have a large refractive index modulation depth (10^−3^) and meanwhile operate stably at 950 °C [141].

In 2004, Martinez et al. first reported direct point-by-point (PbP) inscription of FBG using an infrared femtosecond laser [139]. This method does not require a phase mask plate and a photosensitive fiber, which extremely simplifies the FBG fabrication process. The grating period can be set by changing the translation speed of the fiber along the focus of the beam. In 2005, they investigated the thermal stability of PbP FBG made by femtosecond laser. Experimental results show that FBG works stably at up to 900 °C, with a permanent drop in reflectivity at 1000 °C and erasure above 1050 °C [141]. In 2008, Li et al. fabricated type I and type II FBGs in H_2_-free and H_2_-loaded fibers by 800 nm fs laser and, meanwhile, studied their spectral and annealing properties in comparison. Short-term annealing experiments showed that type II FBGs in H_2_-free fibers have the highest thermal stability, followed by H_2_-loaded type II, H_2_-free type I, and then H_2_-loaded type I FBGs. Long-term thermal stability tests at 1000 °C were carried out on type II FBGs after annealing treatment that was held at 700 °C for 12 h. The result showed that the thermal stability of the H_2_-loaded type II FBG is significantly improved by the high-temperature pre-annealing treatment, and the type II FBG, written in both H_2_-loaded and H_2_-free fibers, maintains a high reflectivity at 1000 °C [136].

**Figure 12:**
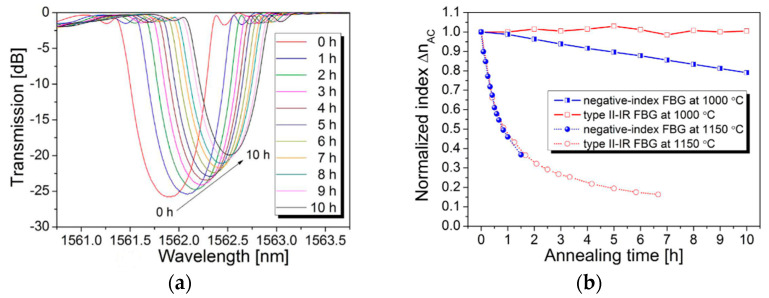
Long-term annealing study of the negative-index FBG. (Reprinted with permission from Ref. [143]). (**a**) Transmission spectra evolution of the negative-index FBG at 1000 °C. (**b**) Evolution of normalized index modulation (Δn_AC_) of the negative-index FBG and the typical type II-IR FBG.

In addition to type I and type II FBGs, there are type IIA FBGs produced by type I FBGs after overexposure with a femtosecond laser [24,142,143], and regenerative gratings produced by type I FBGs after high-temperature heating [144,145]. In 2012, Cook et al. reported a regenerated FBG fabricated by near-infrared femtosecond laser exposure and thermal regeneration treatment [145]. Although the low regeneration rate of the seed grating makes the regenerative grating reflectivity less than 8%, the FBG can operate briefly at 1200 °C. In 2016, He Jun et al. demonstrated a negative refractive index fiber with high reflectivity (99.22%) [143]. During the experiments, the type I-IR FBGs were first overexposed to form polarization-dependent phase-shifted FBGs and then regenerated at 800 °C for 12 h to form negative refractive index FBGs. The thermal stability of negative refractive index FBG is stronger than that of type I-IR FBG and weaker than that of type II-IR FBG. The results of the thermal stability comparison are shown in Figure 12. Compared with type II-IR FBG, the negative refractive index FBG decays slowly at 1000 °C.

In addition, femtosecond lasers can inscribe FBGs in pure silicon fibers with higher operating temperatures. In 2005, Fu et al. first reported the inscription of FBGs in pure silicon photonic crystal fibers (PCFs) using UV femtosecond lasers [146]. However, the FBG is less thermally stable, and the refractive index decreases with the increase in temperature. Moreover, it is found that the air holes in PCF will strongly scatter the femtosecond beam, which is not favorable for type II FBG inscription. In 2016, Warren-Smith et al. fabricated FBGs capable of operating at high temperatures of 1300 °C on silica suspended core fibers (SSCF) [130]. The special geometry of the SSCF facilitates the direct FBG etching of the femtosecond beam through the fiber cladding. The results of the high-temperature tests show that the reflected spectrum remains stable for a holding time (5 min) up to 1300 °C, with permanent damage to the grating when held at 1350 °C for 1 h. Subsequently, they conducted a comparative analysis of the wavelength stability at high temperatures of four types of FBGs: a twisted (chiral) pure-silica microstructure optical fiber [147], a regenerative FBG, a femtosecond laser written Bragg grating in SMF, and a femtosecond laser written Bragg grating in SSCF [148]. The results show that all four types of FBGs can work stably at 900 °C, and two types of FBGs made of pure silicon fiber have better wavelength stability at temperatures above 700 °C.

In 2017, Wang et al. fabricated a small segment of single-clad SSCF using the expanded commercial PCF method and then performed FBG inscription on that segment of SSCF [149]. Experiments proved that the pure silicon FBG made by this method has good stability at high temperatures of 30–1000 °C, and slow erasure occurs when the temperature is higher than 1120 °C.

Table 5 summarizes the above FBGs fabricated with femtosecond lasers in various silica fibers. Researchers have tried various silicon-based materials for FBG fabrication. However, the maximum temperature at which the silicon fiber can survive (1330 °C) limits the upper limit of the temperature measurement of the sensor. Therefore, it is necessary to make high-temperature FBG on other high-temperature resistant fiber to increase the upper limit of temperature measurement.

### 5.2. Sapphire FBGs for High-Temperature Sensing up to 2000 °C

Single-crystal sapphire fiber has a melting point (2045 °C) much higher than that of silica (1330 °C), so it is very suitable for making high-temperature resistant FBG. The current femtosecond laser fabrication of sapphire fiber grating (SFBG) is mainly divided into four types: phase mask technology, Talbot interferometer technology, femtosecond laser point-by-point inscription method, and femtosecond laser line-by-line inscription method. This section will introduce each inscription method and SFBG sensing characteristics.

#### 5.2.1. Phase Mask Method

**Figure 13:**
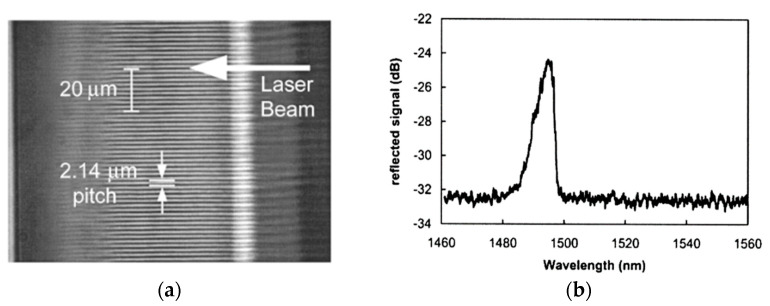
The microscope image and reflection spectrum of SFBG. (Reprinted with permission from Ref. [151]). (**a**) Microscope images of the grating structure written in 150 μm diameter sapphire fiber; (**b**) Reflection spectrum of the SFBG.

Similar to the fabrication of FBGs in silica fibers with the femtosecond laser phase mask technique, ultrahigh peak power fs laser pulse is used to irradiate a sapphire fiber to achieve the permanent refractive index of an optical fiber core. In 2004, Grobnic et al. first reported the fabrication of FBGs using femtosecond laser radiation in multimode crystalline sapphire fibers [151]. The grating structure was written in a 150 μm diameter sapphire fiber by using a 125 fs, 800 nm laser pulse, and a 4.28 μm phase mask. The laser beam will be diffracted after passing through the phase mask and the resulting phase streaks will achieve periodic refractive index modulation. With *Ʌ* = 2.14 μm, the grating is a fifth-order retro-reflecting grating with *n_eff_* = 1.746. Figure 13 shows the microscope image and reflection spectrum of SFBG. The wavelength thermal response characteristics of the SFBG were measured from 22 °C to 1530 °C with a temperature response factor of 25 pm/°C. Sapphire fibers are usually highly multimode because they use air as the refractive index cladding, which results in SFBGs typically having larger half-height widths (~6 nm) and lower reflectivity.

**Figure 14:**
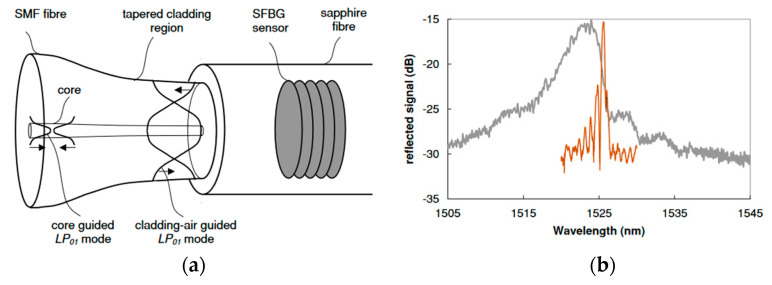
(**a**) Schematic of the tapered fiber coupling method to reduce the spectral width of SFBG; (**b**) Comparison of single-mode (orange line) and multimode (grey line) reflection spectra of the SFBG. (Reprinted with permission from Ref. [152]).

In order to reduce the bandwidth of the SFBG, a tapered fiber coupling method was proposed by D. Grobnic in 2006 [152]. In this method, a single-mode tapered fiber is connected to an SFBG, and a low-loss single-mode (LP_01_ mode) response is obtained from a multimode sapphire fiber by matching the outer diameter of the tapered SMF end and the sapphire fiber. Figure 14a shows the schematic diagram of mode field transmission for the tapered fiber coupling, and Figure 14b shows the comparison of reflection spectra obtained from single-mode tapered fiber coupling and multimode coupling to the SFBG. Although this method can effectively reduce the SFBG half-height width, the coupling method requires high matching accuracy and the fiber coupling position should be far enough away from the temperature measurement point, which is not suitable for extreme environmental applications. In 2007, Zhan et al. shortened the bandwidth of the SFBG by bending a small diameter (60 μm) sapphire fiber [153]. Although a narrow resonant peak bandwidth of 2 nm was finally achieved, the reflection spectrum of SFBG had a poor signal-to-noise ratio.

In contrast to the reduced mode approach described above, in 2009, Busch et al. used a 50 m long multimode fiber as the connecting fiber in an attempt to excite all guided modes in an almost uniformly distributed manner [154]. They finally achieved a high-temperature measurement of 1745 °C with better than 1 °C repeatability and explored the possibility of multiplexing two SFBGs for the first time.

The femtosecond laser phase mask technique for inscribing SFBG has successfully increased the upper limit of FBG temperature measurement. However, the fabrication of phase masks is relatively complicated and can only be used for fixed Bragg wavelength FBG inscription, so it is necessary to develop new techniques that can flexibly inscribe SFBG arrays.

#### 5.2.2. Talbot Interferometer

The Talbot interferometer technique is an optimization in femtosecond laser phase mask technology. Compared to the phase-mask method, the Talbot interferometer method allows easy fabrication of different Bragg wavelength SFBGs without changing the phase mask, providing excellent flexibility and the possibility of making SFBG arrays. The technique utilizes the Talbot interferometer, and adjusting the two rotating mirrors can preset the angle β of the two coherent beams, ultimately achieving a flexible setting of the FBG period [155].

In 2013, Elsmann et al. achieved three SFBGs inscriptions on a single sapphire fiber using Talbot interferometry [156]. The laser pulse (pulse duration: 135 fs, average power: 550 mW, repetition rate: 1 kHz) at an inscription wavelength of λ = 400 nm is launched into the Talbot interferometer for grating inscription. Three SFBGs can be produced by moving the cylindrical lens to scan the focus line through the full diameter with a velocity of 0.1 mm/s. The experimental results showed that the three SFBGs performed well in the sensing range of 100–1200 °C with a sensitivity of 30.1 pm/K. However, the wider reflectance spectrum bandwidth limits the multiplexing capability of SFBG. In 2015, Habisreuther et al. demonstrated a novel high-temperature SFBG sensor [131]. The SFBG was shown to have good temperature measurement performance at 1900 °C, which is the highest measured temperature reported for SFBG experiments so far. In addition, the sensor has a temperature resolution of ±1 K and a response rate of 20 Hz when the temperature is above 1500 °C. These fully demonstrate the potential of the SFBG for applications in high-temperature testing.

After packaging and calibrating, the above SFBG arrays can be used for stable temperature measurements. In 2022, Eisermann et al. developed a hybrid high-temperature sensor combining a thermocouple, blackbody radiation, and SFBG to improve the stability of measuring the wavelength shift of SFBG [157]. The sensor was placed in a fiber draw tower for 3 weeks and over 100,000 measurements were conducted with temperatures up to 1600 °C. Analysis of all data points (100,000) shows that 96% of them fall within this uncertainty, demonstrating the extreme stability of the sensor in harsh environments.

Although the Talbot interferometer method is more flexible than the phase mask method, it requires a guarantee that the path difference between the two interference arms is less than a few microns due to the very short pulses, which increases the operational difficulty of the method.

#### 5.2.3. Point-by-Point Inscription

Compared with the phase-mask method, the femtosecond laser point-by-point SFBG (PbP SFBG) inscription is more flexible and easier to fabricate FBG arrays. In 2017, Yang et al. demonstrated for the first time the fabrication of FBGs on sapphire fibers using femtosecond laser point-by-point inscription and experimentally demonstrated the high-temperature testing capability of three FBG arrays at 1400 °C [158]. The grating (pitch: 1.776 μm, width 1.81 μm, depth 3.28 μm) is inscribed by using a femtosecond laser (emission wavelength *λ*: 780 nm, pulse width: 100 fs, repetition rate: 500 Hz). A 125 μm diameter, 40 cm long sapphire fiber is precisely fixed on a five-axis displacement table, and the SFBG fabrication at different Bragg wavelengths is achieved by adjusting the fiber movement speed and the laser repetition frequency. By adjusting the focal length *f* of the high magnification lens and the input beam waist *w*_0_, a focal area of 0.2 μm diameter is achieved via w = *λf*/*πw*_0_, which satisfies the condition of inscribing a 2 μm pitch grating. The polarization of the incident beam was tuned to be perpendicular to the optic axis of the sapphire fiber through a linear polarizer. The length of the fabricated SFBG is 2 mm, and the total fabrication time is only 2.5 s. Three FBGs were fabricated by adjusting the fiber movement speed along the axial direction. High-temperature heat treatment of SFBG revealed that SFBG shows a permanent enhancement of refractive index at temperatures above 600 °C, with a 5-fold increase in reflectivity at 1400 °C. However, the multimode nature of sapphire fiber still results in a wide reflection spectrum of the fabricated SFBG, which limits the number of FBGs that can be multiplexed.

To reduce the SFBG reflection spectrum width, in 2018, Yang et al. proposed a moist-heat acid etching method to fabricate micro-single crystal sapphire fiber FBG (micro-SFBG) [159]. In this method, a large diameter (125 μm) SFBG is first fabricated using the point-by-point inscription method, and then the SFBG is exothermic in a sulfuric acid-phosphoric acid solution to reduce the fiber diameter. Ultimately, refractive index measurements from 1 to 1.75 and temperature measurements from room temperature to 1400 °C can be achieved using a micro-SFBG with a diameter of 9.6 μm. However, this etching method is time-consuming, which usually takes tens of hours, and the sapphire fiber strength gets weaker with reduced diameter.

Limited by the small refractive index modulation area within the sapphire fiber, the PbP SFBG fabricated by Yang et al. has only 0.6% reflectivity. In 2021, Xu et al. demonstrated a filamentation process to fabricate PbP SFBGs [160]. The principle is to use a single pulse to create an elongated filament trajectory, and the filament trajectory can expand the cross-sectional area of the refractive index modulation, thereby increasing the SFBG reflectivity. They achieved precise control of the filament shape by adjusting the pulse energy and focal depth. After optimizing the parameters, filament trajectories of 90 microns long and 1.4 microns wide were produced. This method takes about 1.1 s to produce SFBG with a grating length of 2 mm and a reflectivity of 2.3%. Subsequently, they successfully fabricated a quasi-distributed sensing array with 5 SFBGs on a 100 μm diameter fiber, which has good temperature measurement performance from 16 °C to 1600 °C.

As the fabrication technology of SFBGs continues to mature, researchers have conducted extensive research into the stability of SFBG high-temperature sensors in practical applications. In 2019, Yang et al. fabricated three SFBGs in a single sapphire fiber using the fs laser via the point-by-point method. The sensor is encapsulated and calibrated and then deployed in a commercial coal-fired furnace and a gas boiler for 42 and 48 days, respectively, to study its long-term high-temperature test performance [1]. Test results show that the sensor is stable over long periods of monitoring at up to 950 °C.

The point-by-point inscription method eliminates the need for phase masks and facilitates the adjustment of grating parameters, greatly increasing the flexibility of fiber fabrication. However, the small area of refractive index modulation formed by a single laser pulse results in only short lengths of fiber grating being written, making it difficult to write highly reflective SFBGs.

#### 5.2.4. Line-by-Line Inscription

To further improve the reflectivity of SFBG, the method of line-by-line etching of FBG (LbL SFBG) in sapphire fiber using a femtosecond laser has been reported successively. The requirements for the size of the focal area and the polarization state of the inscribed beam for the line-by-line inscription method are similar to the point-by-point inscription method. The difference lies in that the fiber to be inscribed moves along both the x-axis and y-axis in the line-by-line inscription method, in which a long refractive index modulation line is inscribed and the refractive index modulation area is increased.

In 2018, X. Xu et al. first proposed and demonstrated the line-by-line inscription of SFBG using a femtosecond laser (wavelength: 524 nm, pulse width: 290 fs, repetition rate: 200 kHz) [161]. By optimizing 3 parameters: fiber diameter, track length L, and grating spacing *ᴧ*, SFBG with reflectivity of 6.3% and bandwidth of 6.08 nm was finally inscribed. By varying the grating pitch, an array containing five SFBGs can be made on a 260 mm long, 60 μm diameter sapphire fiber. High-temperature tests show that the temperature sensitivity of these SFBGs becomes progressively larger with increasing temperature, with a sensitivity of 36.5 pm/°C at 1612 °C. In 2019, Guo Qi et al. similarly realized the fabrication of LbL SFBGs on a 60 μm diameter sapphire fiber and found that SFBGs have a high reflectivity of 15% when the track length is 40 μm [162]. Experimental tests have shown that this SFBG has a sensitivity of 34.96 pm/°C between 1000 and 1600 °C, in addition to a good strain sensitivity.

Furthermore, in order to improve the reflectivity of SFBGs and reduce the reflection spectrum width of SFBGs, in 2019, Xu et al. fabricated a multilayer, bias-coupled SFBG [163]. Compared with single-layer LbL SFBGs [161], two parallel traces are inscribed at the inscription point of each fiber z-axis, and the distance d between the two traces is the layer spacing. The results show that the reflectance of the double-layer SFBG is 34.1% at a layer spacing of 5 μm, which is much higher than that of the single-layer SFBG (6.3%). In addition, the offset coupling of multilayer mode SFBGs to the transmitting multimode fiber reduces the reflection spectrum width by 1.32 nm. This SFBG can still operate normally at 1612 °C with a temperature sensitivity of 45.2 pm/°C.

**Figure 15:**
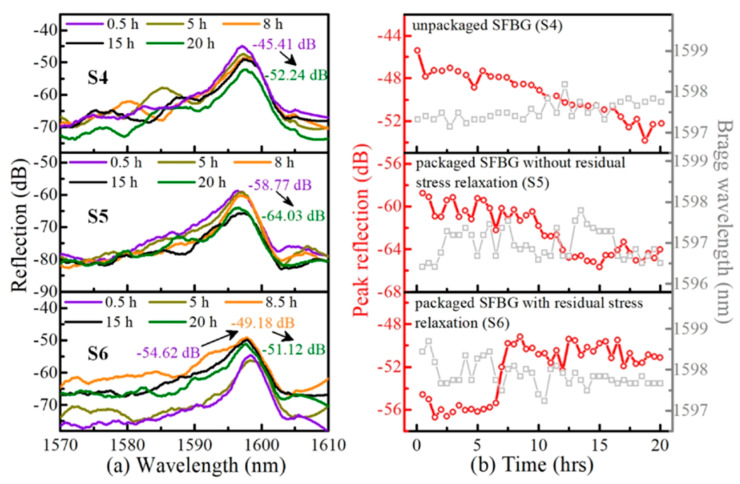
(**a**) Reflective spectral evolutions of SFBG sensors S4–S6 at 1600 °C after 20 h; (**b**) Evolutions of peak reflection and Bragg wavelength of SFBG sensors S4–S6 at 1600 °C after 20 h. (Reprinted with permission from Ref. [167]).

In addition, when sapphire fibers are operated at high temperatures for a long time, some lossy spots and micro-etched lines form on their surface due to high-temperature oxidation [164,165]. Studies have shown that these changes can lead to degradation of the SFBG spectrum and thus reduce the long-term stability of the sensor [166]. In 2022, He et al. effectively overcame these problems by optimizing the fabrication and encapsulation of the SFBG sensor [167]. In the experiments, the isolation of the sapphire fibers from the ambient air using an inert gas encapsulation process inhibited the formation of lossy spots. Moreover, the annealing process with a gradient temperature rise has successfully suppressed the formation of micro-etched lines. The results show that the SFBG could be operated at 1600 °C for 20 h with essentially no change in reflection spectrum shape and SNR, as shown in Figure 15.

In summary, SFBG high-temperature sensors have good development prospects. Table 6 summarizes the characteristics of the different fabrication techniques for the above SFBGs. Optimization of the reflectance spectrum shape and reflectivity of the SFBG is the focus of future SFBG inscription technology, and research into the packaging process for SFBG sensors will also help to promote widespread use in industry.

## 6. Distributed Sensing

Distributed optical fiber sensing (DOFS) systems are widely used for fire warning in railroad tunnels, temperature monitoring of power transmission lines, and health monitoring of oil and gas pipelines [7,168,169,170]. Unlike complex point sensor arrays, DOFS systems require only a single fiber to achieve distributed temperature measurements. The system achieves distributed sensing by demodulating the backscattered signal within the transmission fiber. Depending on the scattering signal (Raman scattering, Brillouin scattering, Rayleigh scattering), DOFS systems with different characteristics can be developed [171,172,173]. The distributed sensor systems based on Rayleigh and Brillouin scattering are sensitive to both temperature and strain, while Raman scattering is the most commonly used distributed temperature measurement system because it is mainly sensitive to temperature. DOFS is generally divided into optical time domain reflection (OTDR) for long transmission distance with low spatial resolution and optical frequency domain reflection (OFDR) [174] for high precision short distance measurement. Suitable distributed sensors can be selected according to the application scenarios [7,169].

### 6.1. Brillouin Scattering-Based Optical Time-Domain Analysis System

The occurrence of photon–phonon inelastic interaction when a pump light is launched in silica optical fibers can lead to generation of a Stokes light with Brillouin frequency shift (BFS) *v*_B_ relative to the pump light. The amount of BFS is usually influenced by the applied temperature and strain that are able to alter the optical and acoustical properties of the optical fibers. Thus, an accurate methodology that can extract BFS values would enable the quantization of the temperature and strain variations. Over the years, various distributed sensing systems based on Brillouin frequency shift measurements have been widely used for temperature and strain sensing [171,173], with Brillouin optical time-domain analysis (BOTDA) being a representative sensing method [7]. In 1990, Kurashima et al. successfully achieved a distributed temperature measurement between −30 and 60 °C for a 1.2 km single-mode fiber by using BOTDA [175]. Since then, most of the reported BOTDA systems have been limited to sensing the ambient temperature using single-mode silica fibers [171].

For high-temperature scenarios in practical applications, single-mode silica fibers are subject to some limitations. Coating burning and dopant diffusion can cause errors in sensing results, especially when scattered light intensity is used as the detection parameter. For example, the burning of fiber-optic coatings at high temperatures (300–500 °C) generates bending losses and compressive stresses that lead to measurement errors [176]. In addition, the transmission properties and mechanical strength of the fiber will deteriorate due to the entry of H_2_ [177] and crack expansion of pre-existing defects on the fiber surface [178]. Recently, it has been reported that the detection of Brillouin frequency shift after high-temperature annealing of SMF is not affected by strength-related factors such as fiber attenuation, splicing, and micro-bending losses [179,180]. For instance, in 2016, Xu et al. conducted distributed Brillouin sensing tests for SMF and PCF at 1100 °C and 1200 °C, respectively [180]. Results demonstrate that silicon fibers can be used for temperature measurements above 1000 °C after primary annealing. However, the mechanical strength of the fiber is significantly reduced and is not suitable for practical industrial temperature measurement applications. In 2017, Ruiz–Lombera et al. demonstrated distributed high-temperature sensing at 600 °C using BOTDA and multimode metal-coated fibers [181]. The sealed metal coating effectively improves the mechanical strength and service life of the sensing fiber at high temperatures; however, the mode coupling of the multimode fiber can lead to sensing errors. In 2018, Xu et al. achieved distributed temperature sensing in a wide temperature range of 1000 °C via BOTDA by using annealed single-mode gold-plated fibers [182]. Currently, there are few reports on BOTDA systems based on single-crystal fiber, as there are many challenges for fabricating long-length single-crystal fibers that can be applied to BOTDA systems with low loss and appreciable nonlinearity indispensable to the inelastic scattering process.

### 6.2. Raman Scattering-Based OTDR System

When a photon undergoes Raman scattering in an optical fiber, the frequency of the scattered photon changes, those below the incident frequency are called Stokes photons and those above the incident frequency are called anti-Stokes photons. The Raman intensities of the anti-Stokes and Stokes components are proportional to their differential cross-sections and are expressed by the following equation [183]:

(9) $${\frac{d\sigma_{AS}}{d\Omega }|}_{x}≅\frac{1}{\lambda_{AS}^{4}}\frac{1}{\exp\left[\frac{hc\Delta v}{K_{B}T\left(x\right)}\right]-1}$$

(10) $${\frac{d\sigma_{S}}{d\Omega }|}_{x}≅\frac{1}{\lambda_{S}^{4}}\frac{1}{1-\exp\left[-\frac{hc\Delta v}{K_{B}T\left(x\right)}\right]}$$

where *h* is Planck’s constant; *K_B_* is Boltzmann’s constant; *λ_AS_* and *λ_S_* are the anti-Stokes wavelength and Stokes wavelength, respectively; *c* is the speed of light in vacuum, *T*(*x*) denotes the temperature at fiber position *x*, and Δ*v* denotes the Raman frequency shift. The intensity of the anti-Stokes component increases approximately linearly with increasing temperature, and the intensity of the Stokes component does not vary essentially with temperature. The temperature *T*(*x*) at fiber position *x* can usually be calculated using the ratio *R* of the two components, *R* is expressed by:

(11) $$R={\left(\frac{\lambda_{S}}{\lambda_{AS}}\right)}^{4}\exp\left[-\frac{hc\Delta v}{K_{B}T\left(x\right)}\right]$$

**Figure 16:**
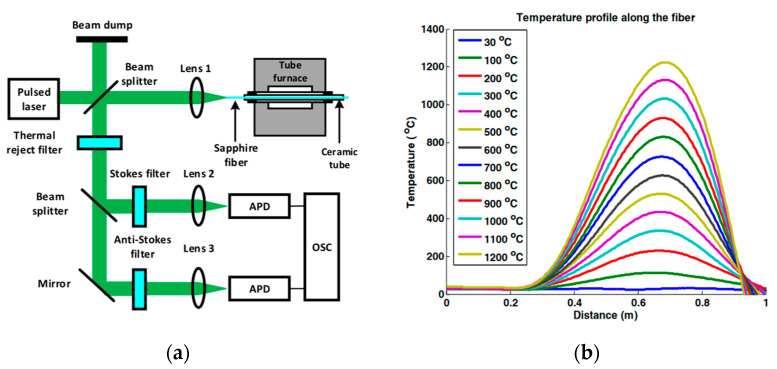
Schematic diagram of the PbP SFBG fabrication process and temperature test results. (Reprinted with permission from Ref. [185]). (**a**) Schematic of the experimental setup for Raman DTS system; (**b**) Demodulated temperature profile along the sapphire fiber.

In 2015, Liu et al. investigated the temperature dependence of Raman scattering peak intensity, frequency shift, and linewidth in sapphire fibers [184]. The intensity change corresponding to the anti-Stokes peak was successfully observed from 300 to 1033 °C. This discovery laid the foundation for the subsequent fabrication of sapphire fiber Raman distributed sensors. In 2016, they achieved the first transition of a Raman distributed sensing system from silica fiber to sapphire fiber [185]. Figure 16a shows the schematic diagram of the experimental setup. Pulsed light with a wavelength of 532 nm (500 μJ) is launched into a 1 m long, 125 μm diameter sapphire fiber, and distributed temperature measurements are achieved using the ratio of the anti-Stokes component to the Stokes component. The short wavelength laser is chosen to avoid blackbody radiation and fluorescent background noise [186,187]. The test results are shown in Figure 16b. The sensor measured the temperature range from room temperature to 1200 °C with an average deviation of 3.7 °C and a spatial resolution of 14 cm. In 2018, they analyzed in detail the impacts of thermal radiation, fluorescence, and multimode nature of single-crystal fiber on this system. After optimizing the system parameters, a spatial resolution of 12.4 cm and position standard deviation of 0.28 mm on a 2 m sapphire fiber was achieved up to 1400 °C [188]. Although the method enables high-temperature distributed measurements, there are still some limitations. The scattered signal is weak, and time averaging is performed to improve the signal-to-noise ratio, which reduces the real-time performance of the system. In addition, an all-fiber structure is necessary to improve the stability and practicality of the system.

### 6.3. Rayleigh Scattering-Based OFDR System

An optical frequency domain reflectometer is a back-reflection technique based on Rayleigh scattering in optical fibers [174]. Light is injected into the reference fiber and the sensing fiber, respectively, and changes in the temperature or strain of the sensing fiber cause changes in the intensity and frequency of the scattered light. Therefore, the spectral offset is obtained by mutual calculation between the reference signal and the measurement signal, and the measurement of external parameters can be realized. Benefiting from ultrahigh spatial resolution, the OFDR system is ideally suited for distributed temperature profile measurements in small areas [189,190]. For example, in 2020, Chen et al. conducted a study inside a fusion splicer by using a 10-mm long single-mode fiber based on OFDR with a spatial resolution of 8 μm and a thermal sensitivity of 10 pm/°C. The results prove that the possible maximum detectable temperature for the optical fiber can reach 2100 °C as generated by an electric arc [189]. Unfortunately, studies of the durability of single-mode fiber under H_2_ flames have shown that the Rayleigh scattering correlation coefficient decreases with increasing temperature, resulting in a short measurement time of only a few seconds for temperatures up to 1100 °C [190]. Since the SNR of distributed sensors based on Rayleigh scattering is mainly determined by the Rayleigh scattering coefficient of optical fibers, ensuring high Rayleigh scattering intensity at high temperatures is the key to achieving long-term stability of distributed high-temperature measurements.

To enhance Rayleigh scattering signals in commercial fiber, UV laser [191,192] and femtosecond laser [193,194,195,196,197,198] micromachining methods have been reported. Monet et al. achieved a 50 dB return signal enhancement using UV laser-induced Rayleigh enhancement cross-sections, which effectively improved the SNR and sensitivity of the OFDR system. However, both the UV laser-induced modifications [192] and the fs laser type I enhancement [194] reported by Mihailov et al. are unstable for long-term high-temperature detection. In 2017, Yan et al. obtained type II nanogratings with greater high-temperature stability using a 300-nJ laser pulse irradiating the fiber at a 250 kHz repetition frequency [193]. Real-time monitoring of solid oxide fuel cell (SOFC) operation at 800 °C with 5 mm spatial resolution has been achieved using the above-mentioned Rayleigh-scattering-enhanced fiber. However, the higher propagation loss of type II modifications limits the spatial scale of distributed sensing. Wang et al. then demonstrated a low-loss, high-temperature stable nanograting reel-to-reel fabrication technique to achieve high-performance distributed temperature measurements up to 1000 °C by using a 3 m long SMF [196,197].

For high-temperature measurements above 1000 °C, it is necessary to change the sensing fiber of the OFDR system to a single-crystal fiber with a higher melting point. However, fiber-optic localization typically utilizes a time-of-flight method that relies on single-mode transmission in the fiber. The multiple mode nature of sapphire fiber can cause time-of-flight distortions and, thus, errors. Although Luna Innovations has utilized multimode fibers for temperature and strain measurements [199,200], there are limitations: (1) the connecting fibers must be highly aligned, (2) multimode fibers cannot be bent.

To expand the OFDR system to sapphire fiber, in 2017, Wilon and Blue successfully fabricated a single-mode sapphire fiber (SMSF) [201]. The cladding was created by irradiating a sapphire fiber, which was surrounded by an annulus of Li-6 enriched lithium carbonate (Li_2_CO_3_) powder. The L36i(n,a)H13 reaction slightly reduces the refractive index around the fiber and forms a cladding inside the fiber. The sapphire fiber (100 μm diameter) after adding the cladding approximates a single-mode fiber. This cladding has a higher survival rate at 1500 °C compared to all the sapphire claddings improved in the literature [166]. Connecting a section of SMSF to a scattering reflectometer (OBR) allows for distributed temperature measurements up to 300 °C. Damage defects caused by fast neutrons may cause irreversible failure when the temperature exceeds 300 °C [201]. Subsequently, they expanded quasi-distributed temperature measurement to 1300 °C by writing five type II Bragg gratings in irradiated single-mode sapphire fiber [202]. The reflection (1–2%) of FBGs is stronger than the fiber intrinsic Rayleigh scattering, which significantly enhances the signal-to-noise ratio of the system.

In 2019, Ohanian achieved distributed measurement of temperature inside a furnace using a 0.55 m long SMSF containing 50 type II FBGs with OFDR interrogation [203]. The system has a spatial resolution of 11mm and is capable of temperature measurements above 1000 °C with an accuracy of 5 °C. Table 7 summarizes the temperature measurement performance of the above DTS systems. In the future, extending the fiber length and inscribing more gratings will enable simultaneous temperature measurement of thousands of data points. The exploration of single-mode sapphire fiber offers the possibility of distributed high-temperature sensing in the future.

## 7. Future Prospects

There is no doubt that single-crystal optical fibers, mainly sapphire fibers, have received a lot of attention in the field of high-temperature sensing. As femtosecond laser micromachining technology and new fiber fabrication techniques continue to advance, the transition of traditional sensing solutions from silica fibers to high-temperature resistant fibers is sure to be a booming area of research. The future development of high-temperature optical fiber sensors faces challenges and opportunities, such as:

1. The development of new high-temperature-resistant optical fiber. The high-temperature resistance of optical fiber is the key to improving the temperature range of the sensor; the preparation of high-quality optical fiber with a high melting point, low loss, strong oxidation resistance, and excellent mechanical properties will greatly promote the development of the high-temperature detection field.
2. Adding cladding to crystal fiber. The lack of a durable cladding limits the sensing performance and long-term stability of crystal fiber sensors. Despite the wide range of crystal fiber cladding materials that have been developed [166], the development and commercialization of crystal fiber cladding remain challenging.
3. Crystal fiber single-mode development. Tapered fiber coupling [152], diameter reduction [159,204], offset coupling [163], and other methods [154], to some extent, overcome the shortcomings of crystal fiber multimode but are less suitable for industrial applications. Radiation [201] or inscribed helical stripes [205] are expected to produce cladding inside the fiber to improve the stability and practicality of the sensor.
4. Multi-parameter measurement. In practice, high-temperature extreme environments are often accompanied by complex state changes. The development of sensors with simultaneous measurement of multiple parameters such as temperature, strain, pressure, and radiation is essential.
5. Long-term stability and package protection. High-temperature sensors are constantly facing the test of high temperature, high pressure, strong radiation, strong corrosion, and other harsh environments, so good package protection to improve the life of the sensor is the goal of the future.

## 8. Conclusions

This paper reviews the development of fiber-optic high-temperature sensors in recent decades. Fiber-optic high-temperature sensors based on blackbody radiation, fluorescence, interferometer, FBG, and scattering processes are introduced separately according to the sensing principle. The transition of the sensing principle from conventional silica optical fiber to crystal optical fiber is described, and a comparative analysis is presented. Table 8 summarizes the temperature range and resolution of the above sensors. The good performance of crystal fiber optic sensors in the field of high-temperature testing and the development prospects are described. Future work should focus on performance optimization of high-temperature-resistant optical fibers and sensor packaging issues. It is believed that fiber-optic high-temperature sensors are expected to replace the traditional electronic thermometers that are currently being widely used in industry.

## Figures and Tables

**Table 1:** Performances comparison of blackbody OFTs.

Blackbody Material	Measurement Range (°C)	Performance	Ref.
Al_2_O_3_-Ir	600–2000	4.6%	[31]
Al_2_O_3_-Cr^3+^	20–1800	Resolution: 1 °C	[30]
BeO	1200–2300	Accuracy: 4%	[37]
Ir	800–1600	-	[38]
MO	900–1800	Deviation: 5 °C	[39]

**Table 2:** Performances comparison of fluorescence-based sensors.

Test Method	Sensing Materials Doped with Rare Earths	Temperature Range	Sensing Performance	Ref.
FL	YAG: Tm^3+^	0–1400 °C	±5 °C	2003 [46]
	YAG: Cr^3+^	−20–500 °C	1 μs/°C @500 °C	2006 [49]
	YAG: Cr^3+^	−25–50 °C	0.1 °C	1995 [50]
	YAG/KGW/YVO_4_: Nd^3+^	0–1000 °C	±2 °C	1997 [51]
	YAG: Yb^3+^	1600 °C	3 °C	2002 [52]
	YSZ/YAG: Dy^3+^	0–1200 °C	-	2009 [53]
	YAG: Dy^3+^, Er^3+^	24–1700 °C	10 °C	2020 [54]
FIR	SiO_2_/YAG: Tb^3+^	300–1200 K	-	2006 [55]
	YAG: Pr^3+^	293–573 K	0.0025 K^−1^	2016 [56]
	YAG: Yb^3+^	500–1000 K	0.3% K^−1^	2018 [57]
	YAG: Sm^3+^	303–1028 K	3.046 × 10^−4^ K^−1^	2022 [58]

**Table 3:** Comparison of micro-structured fiber-optic interference sensors based on silica fiber.

Sensor Structure	Microstructure Type	Fiber Type	Operating Temperature (°C)	Sensitivity	Ref.
MZI	Taper	HF	200–1000	12 pm/°C	[69]
		SMF	20–400	0.07 nm/°C	[70]
	Diameter mismatch	SMF-MMF	0–900	0.088 nm/°C	[71]
		SMF-TCF	0–850	18.3 pm/°C	[72]
	Trench structure	SMF	200–875	51.5 pm/°C	[73]
		SMF	100–1100	0.046 nm/°C	[74]
	Fs laser	SMF	500–1200	109 pm/°C	[75]
		SMF	0–1100	41 pm/°C	[76]
FPI	Fusion splice	PCF-HOF-SMF	0–1000	-	[77]
		SMF-PCF	25–1200	17.2 nm/°C	[78]
		SMF-PCF	25–1200	10 pm/°C	[79]
		SMF-MF	25–1000	13.6 pm/°C	[80]
		SMF-HCF	0–1000	12.26 pm/°C	[81]
	Dielectric film	MMF	250–750	5.4 pm/°C	[82]
	Fs laser	SMF	25–1100	0.074 pm/°C	[83]
		SMF	25–1100	9.91 pm/°C	[84]
		SMF	25–1100	10.15 pm/°C	[85]
	Silicon integration	SMF	120–400	106 pm/°C	[86]
MI	Diameter mismatch	SMF-MMF	100–750	15 pm/°C	[88]
		SMF-MCF	250–600	165 pm/°C	[91]
	Taper	SMF-TCF	30–800	140 pm/°C	[94]
		SMF	100–800	166 pm/°C	[97]
	Reflector	SMF	50–1000	14.72 pm/°C	[101]
		SMF	400–1000	68.1 pm/°C	[102]

**Table 4:** Comparison of sapphire interference sensors.

Sensor Structure	Fiber Diameter (Μm)	Operating Temperature (°C)	Performance	Ref.
SMF-SPF	125	310–976	0.2 °C	1992 [104]
	125	256–1510	0.1 °C	1992 [105]
Air cavity	-	25–650	3.5 °C /10 με	1995 [106]
	100	0–1004	0.2 με @1004°C	2003 [107]
	75	0–1050	0.3 °C (multiplexed)	2010 [108]
Sapphire wafer	75	230–1600	±0.2%	2005 [109]
	75	24–1170	0.4 °C	2006 [110]
	-	25–800	1.25 nm/°C and 0.00025 nm/(kPa·°C)	2019 [112]
Ta_2_O_5_	75	200–1000	1.4 °C	2011 [113]
ZrO_2_	-	200–1000	5.8 °C	2012 [114]
ZrO_2_/Al_2_O_3_/ZrO_2_	-	100–1111	1.8 × 10^−5^/°C	2015 [115]
OCMI	-	100–1400	64 kHz/°C	2015 [122]

**Table 5:** Comparison of silica FBGs sensors.

Fiber	Grating Type	Treatment	Performance	Ref.
SMF	Type I	None	Stable to ~300 °C	[137]
SMF	Type II	Residual stress relaxing by pre-annealing of the SMF	Stable to ~1200 °C, easily brittle	[150]
SSCF	Type II	None	Stable to ~1300 °C	[130]

**Table 6:** Comparison of SFBGs fabrication techniques.

Technology	Operating Temperature (°C)	Sensitivity	Advantages	Disadvantages	Ref.
Phase mask	22–1530	25 pm/°C	Easy mass production	Difficult to make FBG arrays	[151]
Talbot	25–1200	30.1 pm/K	Flexible	High accuracy requirements	[156]
Point-by-point	25–1400	25.8 pm/°C	Simple, flexible	Low reflection (0.6%)	[158]
Line-by-line	25–1612	36.5 pm/°C	High reflection (6.3%)	Complicated operation	[161]

**Table 7:** Comparison of distributed sensors.

Technology	Fiber Type	Treatment	Sensing Range	Spatial Resolution	Temperature Accuracy	Temperature Range	Ref.
Brillouin OTDA	SMF, PCF	Annealing	50 m @SMF 2 m @PCF	1 m	2.4 °C @SMF 3.6 °C @PCF	Above 1000 °C	[180]
	Gold-coated fiber	None	1 m	20 cm	1.35 MHz/°C	Stable to ~1000 °C	[182]
Raman OTDR	Sapphire fiber	None	2 m	12.4 cm	3.7 °C	Stable to ~1400 °C	[188]
Rayleigh OFDR	SMF	Nanograting	1 cm	3 m	0.012 °C	Stable to ~1000 °C	[197]
	Sapphire fiber	Inside cladding, type II FBG	11 mm	0.55 m	5 °C	Stable to ~1000 °C	[203]

**Table 8:** Summary of single-crystal optical fiber sensors for high-temperature measurement.

Sensor Type	Operating Temperature (°C)	Performance	Advantages	Disadvantages	Ref.
Blackbody radiation	900–1800	Resolution: 5 °C	Rapid response, low cost	Small temperature detection range, needs calibration	[39]
Fluorescence-based	24–1700	Resolution: 10 °C	Simple fabrication, low cost	Poor linearity, needs calibration	[54]
FPI	230–1600	Accuracy: ±0.2%	High accuracy, flexible design	Easy contamination, low SNR	[109]
MI based OCMI	100–1400	Resolution: ±0.5 °C	High SNR	Complicated operation	[121]
FBG	25–1900	Resolution: ±2 K	Absolute measurement, easy to multiplex	High cost, complicated fabrication	[131]
Distributed	25–1400	Deviation: 3.7 °C	Distributed measurement	High cost, not all-fiber structure	[188]

## Data Availability

Not applicable.
